# Functional analyses and single cell immunoprofiling uncover sex-specific differences in SARS-CoV2 immune memory development

**DOI:** 10.21203/rs.3.rs-1416969/v1

**Published:** 2022-03-15

**Authors:** Basak Eraslan, Eric Brown, Maura Benson, Liat Amir-Zilberstein, Sung-Moo Park, Betsabeh Tusi, Vladislav Pokatayev, Cody Hecht, Novalia Pishesha, Devan Phillips, Andy Kim, Shuting Zhang, Anthony Gaca, Fadi Ghantous, Toni Delorey, Jonathan Livny, Lindsey Baden, Orit Rozenblatt-Rosen, Daniel Graham, Aviv Regev, Michael Seaman, Ann Woolley, Lisa Cosimi, Deborah Hung, Jacques Deguine, Ramnik Xavier

**Affiliations:** Broad Institute of MIT and Harvard; Broad Institute of MIT and Harvard; Brigham and Women’s Hospital; Broad Institute of MIT and Harvard; Massachusetts General Hospital, Harvard Medical School; Massachusetts General Hospital; Broad Institute of MIT and Harvard; Broad Institute of MIT and Harvard; Broad Institute of MIT and Harvard; Broad Institute of MIT and Harvard; Brigham and Women’s Hospital; Broad Institute of MIT and Harvard; Broad Institute of MIT and Harvard; Beth Israel Deaconess Medical Center; Broad Institute at MIT; The Broad Institute of Massachusetts Institute of Technology and Harvard University; Brigham and Women's Hospital; Broad Institute of Harvard and MIT; Broad Institute; Broad Institute of MIT and Harvard; Beth Israel Deaconess Medical Center; Brigham and Women’s Hospital; Brigham and Women’s Hospital; Broad Institute of MIT and Harvarad; Broad Institute of MIT and Harvard; Broad Institute of the MIT and Harvard

## Abstract

SARS-CoV-2 infection leads to a broad range of outcomes and immune responses, with the development of neutralizing antibodies generally correlated with protection against reinfection. Here, we have characterized both neutralizing activity and T cell responses in a cluster of subjects with mild disease linked to a single spreading event. Surprisingly, we observed sex-specific associations between spike- and particularly nucleoprotein-specific T cell responses and neutralization, with pro-inflammatory cytokines being linked to higher titers only in males. Using single cell immunoprofiling, which provided matched transcriptome and T-cell receptor (TCR) profiles in restimulated CD4 + and CD8 + cells from these subjects, we identified differences in type I IFN signaling that may underlie this difference in antibody generation. Finally, we also identified several TCRs associated with cytokine producing T cells. Altogether, our work maps the breadth of immunological outcomes of SARS-CoV2 infections and highlight the potential role of sex-specific feedback loops during the generation of neutralizing antibodies.

## Introduction

The coronavirus disease 2019 (COVID-19) pandemic has led to more than 400 million cases and 5 million deaths confirmed as of early 2022 (www.who.int/covid-19). Over the past year and a half, many studies have helped characterize the biology of SARS-CoV-2 and the role of the immune system in both pathogenesis and protection, and have notably led to the development of multiple highly effective vaccines to SARS-CoV-2 as well as some therapeutic options including recombinant neutralizing antibodies (reviewed in ^1^).

In addition to these therapeutic advances, the large-scale immunoprofiling of COVID-19 subjects has offered important insights into the basic immunobiology of responses to acute infections. COVID-19 severity has been associated with distinct cytokine profiles and cellular compositions ^2-5^, generally fitting with the notion of a “cytokine storm” that contributes to pathology and have demonstrated an expected network across immune cell states. Beyond these studies of acute cases, several groups have profiled the memory responses in convalescent individuals, with an initial focus on identifying neutralizing antibodies developed during infection ^6-8^ and on the SARS-CoV-2 epitopes recognized by memory T cells ^9-11^. Of particular relevance here, the application of single cell sequencing technologies has allowed the joint mapping of T and B cell receptor (TCR/BCR) sequences and transcriptional profiles ^8,12^, while larger scale studies have mapped TCR repertoires more broadly ^13,14^. In spite of these studies, the link between epitopes, TCRs and T cell function, as well as its impact on the development of antibody responses, remains only partially understood.

Here, we leveraged an existing cohort of convalescent subjects, where we had previously profiled antibody responses ^8^, to combine functional analyses of spike and nucleoprotein re-stimulated T cells with single cell sequencing technologies. We observed a diverse array of T-cell cytokines produced in response to SARS CoV-2 antigen – both spike and nucleoprotein – including those associated with Th1, Th2 and Th17 cells. T cell responses to nucleoprotein were a better predictor of previous infection compared to spike protein. Surprisingly, we identified a sex-specific association between T cell cytokine production and the development of neutralizing antibody responses. Upon profiling of the T-cell responses using scRNA-seq, we observed a number of genes upregulated in a sex specific manner in SARS CoV-2 responding CD4+ T-cells, including those involved in the type 1 interferon response. In addition, scRNAseq analyses of re-stimulated cells highlighted a broad functional diversity of SARS CoV-2 responding T-cells that is not observed in baseline PBMCs, and links TCR sequence to specific functional profiles.

## Results

### Cohort of previously SARS-CoV-2-infected individuals

**Figure 1:**
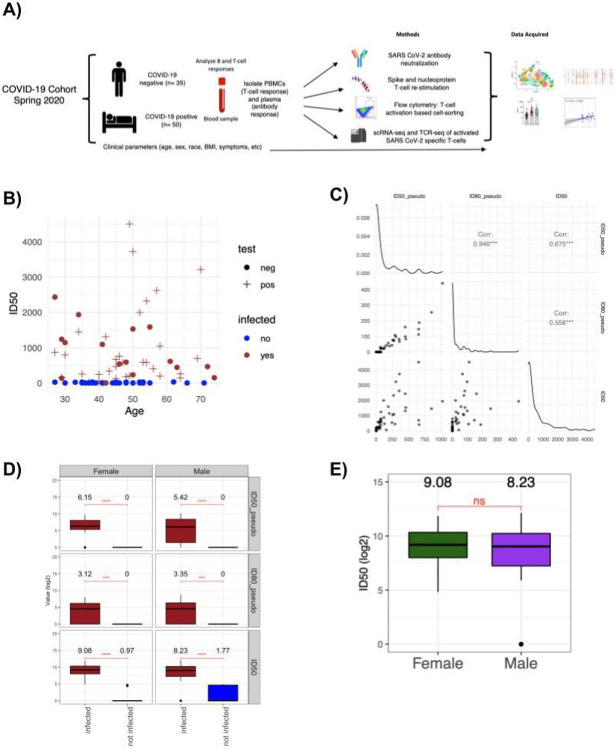
Heterogenous development of antibody responses in a mild COVID-19 cohort **A)** Experimental approach. 85 subjects were recruited from a single spreading event, and PBMCs and serum samples were collected for analyses of antibody responses, T cell activation assays and single-cell RNAseq. **B)** ID50 levels in an authentic SARS-CoV2 neutralization assay for each subject. Infection status was evaluated based on a PCR positive test (plus signs) or a negative test / absence of test (circles), as well as serology (red indicates a positive result for IgG spike, blue indicates negative). **C)** Scatterplots displaying the correlations between different neutralization assays, using a pseudotyped virus (ID50_pseudo and ID80_pseudo for titers neutralizing 50% or 80% of infection) or authentic SARS-CoV2 (ID50). Diagonal plots display the density of the values. **D)** Boxplots displaying the distribution of the ID50_pseudo, ID80_pseudo and ID50 neutralization titer values of the subjects stratified by their sex and infection status. The boxes represent −2 standard deviation (lower portion), mean (black line), and + 2 standard deviation (upper portion). The values above each violin plot represent the median values of the distribution. Brackets indicate statistical significance using a one-sided t-test with **P ≤ 0.01, ***P ≤ 0.001, ****P ≤ 0.0001 and ns=non significant. **E)** ID50 neutralizing titers against authentic SARS-CoV2 across male (purple) and female (green) subjects. There is no significant difference between the means of the two groups based on a two-sided t-test.

To investigate and characterize the development of immune memory after COVID-19, we obtained samples from a cohort of 85 individuals that were either infected or close contacts of SARS-CoV-2 infected subjects during the early months of the pandemic in the U.S Northeast. All symptomatic individuals included in the cohort had a mild disease course (1–2 on W.H.O. severity scale). Of these subjects, 38 were men and 47 were women, with an average age of 46 (Table 1). Samples were collected in the 5–6 weeks that followed the onset of symptoms or a potential infectious contact to evaluate the development of antibody responses as well as the phenotype of memory T cells generated during this infection. To this end, we collected both serum and whole blood, and PBMCs were isolated from whole blood within 4 hours of blood draw and cryopreserved. In addition, participants filled out a survey reporting clinical information and including the duration and nature of their symptoms (Table 1; Fig. 1A).

Out of 85 individuals, 34 were confirmed positive by nasopharyngeal swab and PCR. However, given the low availability of testing during the first wave of infections, it seemed likely that some individuals were infected but not tested, or tested after their SARS-CoV-2 viral load became undetectable. We therefore performed serology testing for both IgM and IgG against the receptor binding domain (RBD) of the spike protein or against the nucleoprotein of SARS-CoV2 on all serum samples from this cohort. As expected, all PCR-confirmed subjects were confirmed positive by serology, with the exception of two individuals that did not appear to mount detectable antibody responses. In addition, we identified an additional 17 individuals who tested positive by serology for responses against RBD (red circles in Fig. 1B), and all but one had demonstrable neutralizing responses (*vide infra*). Ultimately, we treated all subjects with either a positive PCR test or positive serology as “previously infected”, and all other individuals as uninfected controls, and we will use these designations hereafter (Table 1).

### Development of SARS-CoV2 neutralizing antibodies

Having established a serological assay, we focused on measuring neutralizing antibodies in serum through two parallel approaches; one using a virus pseudotyped with the SARS-CoV-2 spike protein (reported as ID50_pseudo and ID80_pseudo, the serum titers that neutralize 50 or 80% of the infection) and another using an authentic SARS-CoV-2 infection model (reported as ID50). In both assays, titers obtained from the two approaches were highly correlated (Pearson = 0.675, p < 0.001 for ID50_pseudo and ID50) (Fig. 1C), and the presence of significant neutralization activity was only detected in infected subjects (Fig. 1D). We noted that neutralization assays with authentic SARS-CoV-2 virus provided a much wider dynamic range (with ID50s ranging from 20 to ~ 3000, versus 20 to 1000 for pseudotyped virus). Based on the high correlation between the two assays, this discrepancy in dynamic range is more likely due to technical considerations rather than the biological impact of non-spike targeting neutralizing antibodies, although this would need to be examined in more detail for further confirmation. Nevertheless, we chose to focus on the neutralization titers generated through authentic SARS-CoV-2 assays through the rest of this study, and focused on understanding the correlates of protection in this cohort.

Next, we examined whether any of the clinical parameters collected were associated with the development of neutralizing activity. In spite of the association between age, sex and disease severity as well as symptom duration, we did not observe any correlation between these parameters and neutralizing antibody titers (Fig. 1E and Supp Fig. 1A-B). As all COVID-19 cases followed here were mild disease by W.H.O criteria, we focused on symptom duration as a surrogate marker of disease course, but again we did not observe any correlation between this parameter and the development of neutralizing antibodies (Supp Fig. 1A). Altogether, these results show that there is a marked diversity in the development of neutralizing antibodies, that does not appear to be correlated with disease course but is probably the result of the inherent heterogeneity of the immune response across subjects.

### SARS CoV-2 specific T cells produce a range of cytokines upon restimulation

To understand how the development of T cell memory and the generation of neutralizing antibodies are correlated after SARS-CoV2 infection, we activated PBMCs from COVID-19 recovered individuals and uninfected controls with a tiled mix of 15mer peptides in a pool, derived from either the spike (S) or nucleoprotein (N) of SARS-CoV-2 (commercially available; see methods). In total, we measured the T-cell cytokine responses across 78 individuals (48 infected and 30 uninfected controls). After restimulation, supernatants were collected and the levels of 10 cytokines capable of being produced by T cells (IL-2, IL-5, IL-6, IL-9, IL-10, IL-17A, IL-17F, IL-22, IFN-γ and TNF-α) were measured with a bead array. First, we examined the presence of SARS-CoV-2 specific responses across the entire cohort, by comparing the levels of cytokines in stimulated and unstimulated cells. We observed a significant induction (FC > 2; p < 0.05, Kruskal-Wallis One-Way ANOVA test) of IL-2, IL-5, IL-17A, IL-17F, IL-22, IFN-γ and TNFα in SARS-CoV-2 infected individuals compared to the uninfected controls (Supp Fig. 2A-B). Of note was the breadth of the cytokine response across SARS CoV-2 infected individuals, and activation of cytokines independent of the canonical antiviral cytokines (e.g. IL-5, IL-17A, IL-22). Among all individuals tested, there was generally a higher amplitude of responses induced by the S-derived peptide pool than by N-derived peptides. This response after restimulation is likely resulting from SARS CoV-2 specific T-cells given the lack of responses in IL-2, TNF-α and IFN-γ we observe in spike restimulated, pre-pandemic PBMCs collected before 2020 (Supp. Figure 2C). Of note, the nucleoprotein peptide mix did include more total 15mer peptides compared to the spike mix (see methods). Conversely, IL-10 was found to be less specific to infected individuals and additionally, found to be upregulated even in PBMCs from uninfected individuals (collected before 2020), suggesting that our approach does restimulate cross-reactive cells described previously ^15^ but that their response can be functionally separated from the responses associated with recent SARS-CoV2 infection (Supp Fig. 2D). We did not observe the induction of IL-4, IL-13 or IL-9 above background levels, and excluded them from further analyses.

**Figure 2:**
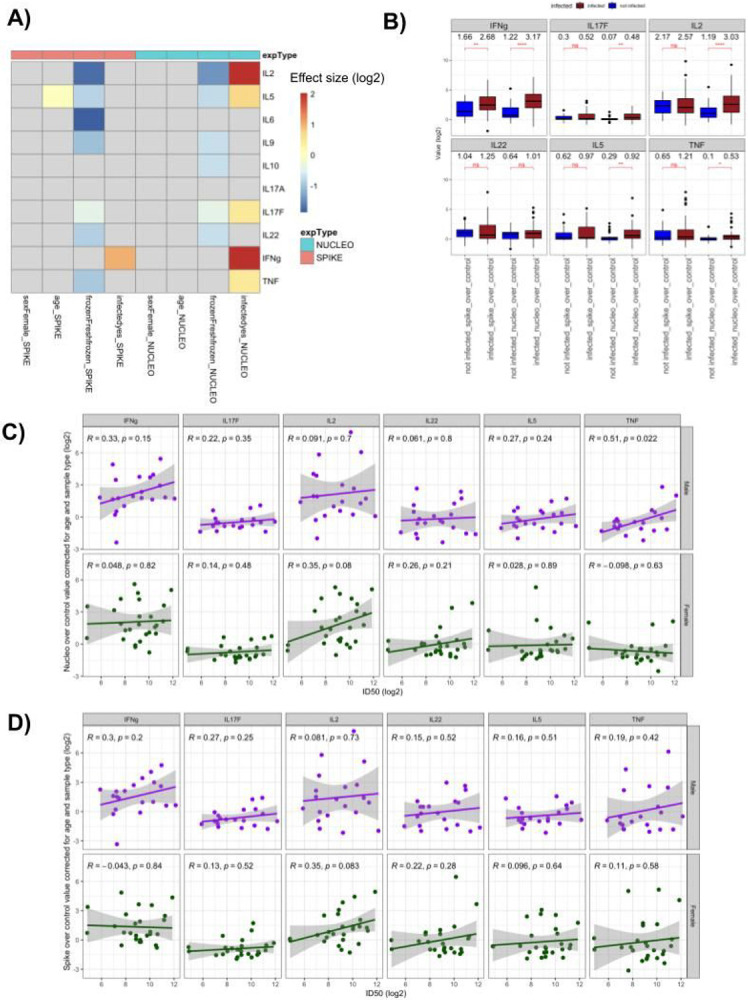
Multiple cytokines are associated with infection and show a sex-specific correlation to neutralizing antibodies **A)** Heatmap displaying the log2 effect sizes of biological (sex, age and infection status) and technical (fresh vs frozen processing) factors on cytokine responses measured as fold change after restimulation with a spike (red, left columns) or nucleoprotein (blue, right columns) peptide pool (Methods). Nonsignificant values (FDR ≥ 0.1) are indicated in gray, significant values are colored by the effect size value in log2 scale. **B)** Box-plots displaying the distribution of IFNγ, IL-17F, IL-2, IL-22, IL-5, and TNFα level fold changes upon spike and nucleoprotein stimulation, stratified by the infection status of the patients (red infected, blue non infected). Brackets indicate statistical significance using a one-sided non-parametric Wilcoxon test with *P ≤ 0.05, **P ≤ 0.01, ***P ≤ 0.001, ****P ≤ 0.0001 and n/s indicating no statistical significance. **(C-D)** Scatterplots displaying the correlation between log2 fold change values of indicated cytokines after nucleoprotein **(C)** or spike **(D)** restimulation corrected for subject age and sample type (fresh or frozen) (y- axis) and neutralization titers (ID50, x-axis) for infected male (top row, purple) and female (bottom row, green) subjects.

To understand the development of SARS-CoV-2 specific responses after infection while considering other factors that can influence cytokine levels, we used a multivariate linear regression model which estimates the possible effects of age, sex, infection status and processing (fresh or cryopreserved cells) over cytokine induction (i.e. log fold change over unstimulated cells) (Fig. 2A-B, Supp Fig. 2E, Methods). As expected, we observed a significant increase in IFNγ production in response to S-peptides and infection status (99% increase, FDR < 0.1, Methods). Perhaps surprisingly given the overall lower responses to N-peptides, we observed a greater number of associations between the cytokine inductions after restimulation with the N-peptide pool and the infection status of the donors. IL-2, IL-5, IL-17F, IFNγ and TNFα inductions were positively associated with the infection status in the N-peptides restimulated cells (308%, 65%, 39%, 304% and 40% increase when infected, respectively, FDR < 0.1, Fig. 2A-B, Supp Fig. 2E, Methods). This result suggest that a broader variety of cytokine responses, potentially corresponding to distinct T helper lineages, are induced by nucleoprotein derived antigens. Notably, in spite of their well-documented influence on disease severity and of the reported effect of age on the nature of immune responses in acute patients ^16^, we did not observe any significant influence of age and sex on the amplitude of cytokine responses, with the exception of a modest association between age and IL-5 production in response to spike antigens (2% increase, FDR < 0.1, Fig. 2A, Methods). From a technical perspective, the processing of the sample as fresh vs. cryopreserved (i.e. frozen) did impact cytokine production and frozen samples generally lowered responses, although the magnitude of this effect depends on the cytokine readout. For example, IL-2 and IL-6 levels were strongly affected (70% and 63% decrease for IL-2 in spike and nucleoprotein respectively, 73% decrease for IL-6 in spike, FDR < 0.1, Fig. 2A, Methods) while IFNγ, and IL-17A were not statistically different between freshly stimulated and previously frozen cells (Fig. 2A, Methods), highlighting the importance of regressing this parameter out of downstream analyses.

### Sex-specific correlations between neutralizing antibody responses and T cell memory

Having characterized the scope of memory T cell responses against SARS-CoV-2, we next focused on the association between levels of neutralizing antibodies and the nature of anti-spike and anti-nucleoprotein T cell responses. Across all infected individuals, we noticed modest correlations between ID50 and cytokine levels for the large majority of the cytokines associated with infection status, as would be expected since ID50 values and multiple cytokine levels are both directly linked to the infection status of the subject (data now shown). To avoid this confounding factor and directly reveal whether specific T cell responses are associated with the production of neutralizing antibodies during infection, we therefore focused only on infected subjects, excluding one individual who tested positive by PCR but showed no antibody or T cell responses above background. Strikingly, when this dataset was broken down across sex, we observed positive correlations between neutralizing titers and inflammatory cytokine production after nucleoprotein restimulation in male subjects (TNFα, R = 0.51, p = 0.022, and IFNγ, R = 0.33, p = 0.15), but these associations were not observed in female subjects, with only IL-2 showing a trend towards association (R = 0.35, p = 0.08) and inflammatory cytokines showing no association at all (TNFα, R = −0.098 and IFNγ, R = 0.048) (Fig. 2C). Similar trends were observed in an independent restimulation to a spike peptide pool, albeit to a lesser degree (Fig. 2D). As noted above, this was not caused by differences in overall neutralizing titers or cytokine production across men and women, since these were similar (Fig. 1E and Fig. 2A), suggesting that the coupling of neutralizing antibody production and memory T cell generation occurs differently across males and females: in males specifically, higher neutralizing antibodies appear to be associated with more type 1 responses, a phenomenon that is not observed in females. Interestingly, when we looked at associations between T cell cytokine responses and symptom duration, as a surrogate marker of disease severity in these clinically mild cases, the only significant association was detected in female subjects, between the levels of TNF elicited after spike stimulation and symptom duration (Supp. Figure 2F-G). Altogether, this suggests that differences in the orchestration of the immune response between male and female subjects can influence the development of neutralizing antibodies, independently of the general strength of the T cell response or of disease course.

### Enrichment and single cell analysis of SARS-CoV-2 responsive T cells

**Figure 3:**
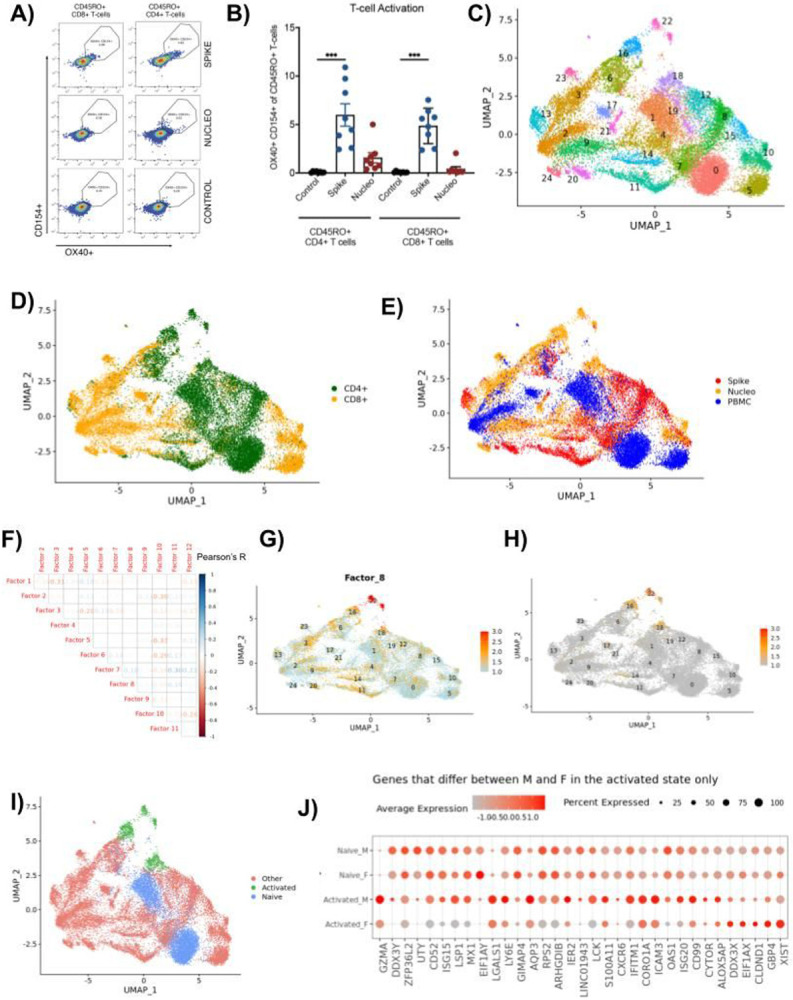
Single cell analysis of steady state and spike or nucleoprotein re-stimulated T cells A) Representative FACS plots of each PBMC stimulation (control vs spike peptides vs nucleoprotein peptides) that was analyzed for SARS CoV-2 specific T-cell responses (n=8). The black circle represents the cells that were sorted and sent for sequencing (OX-40 and CD154 positive), which we call activated cells. Plots are showing gated CD4+ and CD8+ T-cells from PBMCs. B) A box plot of the percentage of cells that are activated based on OX-40 and CD154 staining of re-stimulated PBMCs from recovered COVID subjects (n=8). CD4+ and CD8+ T-cells are shown restimulated with spike (blue) and nucleoprotein (red). Mann Whitney *U*-test (***p<0.001). **C)** Uniform manifold approximation and projection (UMAP) plot displaying the clusters on the 2D projections of the cells based on their scRNAseq transcriptome profiles. **D)** Same as C, cells are colored based on whether they are CD4+ or CD8+ cells. **E)** Same as C, cells are colored based on whether they belong to PBMC, spike restimulated, nucleoprotein stimulated samples. **F)** Pearson correlation coefficients between the 12 latent factors that generate the main axes of variation in the transcriptome of the 25,167 cells collected from spike and nucleoprotein stimulated and PBMC samples. **G)** Same as C, cells are colored based on the factor 8 scores. **H)** Same as C, cells are colored based on their cytokine expression scores (Methods). **I)** Same as C, cells are colored based on whether they belong to the clusters associated with high cytokine production (TCs 16, 18 and 22, green), or to the clusters of resting PBMC states (TCs 0 and 1, blue) or to other clusters (red). **J)** Dot plot displaying the scaled expression levels of genes that are significantly differentially expressed (FDR < 0.1, absolute log2FC > 0.4) between male and female subjects only in the activated (TCs 16, 18 and 22) group but not in the naive group (TCs 0 and 1).

To follow up on this observation and try to define the molecular underpinnings of this difference in immune response orchestration, we decided to analyze the phenotype of SARS-CoV-2 specific T cells by single cell RNA sequencing (scRNAseq). To be able to broadly analyze T cells across all HLA types to multiple SARS CoV-2 epitopes, we designed a strategy based on the isolation of activated T cells after restimulation with a pool of peptides from the spike protein and nucleoprotein. As a first validation, we measured the upregulation of activation markers CD154 and OX-40 on CD4 + and CD8 + T-cells across 8 previously infected individuals after restimulation with spike and nucleoprotein pools, using flow cytometry (Fig. 3A). In previously infected donors, a significant fraction of cells expressed the activation markers CD154 and OX40, suggesting that these markers can be used to enrich SARS-CoV2-specific T-cells (Fig. 3B). From these data, we also observed a higher percentage of CD4 + and CD8 + T-cells were activated upon re-stimulation with the spike protein compared to the nucleoprotein (Fig. 3B).

Using this sorting strategy, we activated and sorted CD8 + and CD4 + T cells activated with either spike or nucleoprotein peptide pools and performed 5’ scRNAseq pairing transcriptome analysis and TCR sequencing. After quality control, we obtained 18,436 cell profiles from 17 subjects across all conditions, with 15,025 of these cells also having a paired fully identified TCR sequence (Supp Fig. 3A). To complement this dataset, we also extracted CD8 + and CD4 + T cells sequenced directly from PBMCs of the same subjects, representing an additional 10,141 cells with complete TCR sequences.

As a first step, we performed Leiden clustering of the resulting dataset to identify the phenotypic profiles of the spike and nucleoprotein restimulated and the unstimulated PBMC cells (Methods). We identified 25 transcriptional clusters (TC) across all CD4 + and CD8 + T cells (Fig. 3C, Supp Fig. 3B, Methods). As expected, clusters derived from CD4 + and CD8 + T (Fig. 3D, Supp Fig. 3C, D) cells were generally well separated, although some clusters did contain cells from both populations (e.g. TCs 4 and 6, that likely represent a signature of cell stress characterized by an enrichment in ribosomal proteins and NEAT respectively, Supp Fig. 3B). Overall embedding structure of the CD4 + cells displayed the cells on the axis of variation representing the naïve to activated states differentiation (Supp Fig. 3E,F, Methods). Likewise, CD8 + cells were positioned based on their naïve to cytotoxic cell states (Supp Fig. 3G,H, Methods). Importantly, cells derived from unstimulated PBMCs clustered independently in a small number of subsets (Fig. 3E, Supp Fig. 3I,J), representing naïve CD4 + and CD8 + cells (Fig. 3C,E, TCs 0 and 5 respectively), T helper cells (TC 1), and two subsets annotated as cytotoxic T cells (TC 2) and a small cluster of CXCR6 + cells (TC 17). By contrast, cells derived from restimulated samples (Fig. 3E, spike in red, nucleoprotein in yellow, Supp Fig. 3I, J) showed a much higher phenotypic diversity that was divided across the remaining 20 clusters. The majority of clusters represented shared states observed across multiple subjects, but we also noted the presence of smaller donor-specific clusters among restimulated cells (in particular TCs 11 and 20 derived from subject 257, Supp Fig. 3K, L). This outlier may reflect specific states that arise during activation, and it is interesting to note that this population was recovered from both CD4 + and CD8 + T cells in this subject, but it may also be linked to the fact this subject was also an outlier in terms of age.

In order to functionally map these clusters, we extracted biological factors with non-negative matrix factorization (Methods), thus defining 12 latent factors that generate the main axes of biological variation across these T cells (Supp Fig. 4A-W). These 12 factors mostly did not correlate with each other, possibly because they stand for independent processes that generate the overall transcriptome variation (Fig. 3F, max Pearson’s R = 0.3, Methods). We then relied on the top genes in each factor to associate these factors with relevant facets of T cell biology (Supp Fig. 4W). For example, factor 10 was highly enriched in non-restimulated cells, and included *SELL, IL7R, LEF1* and *TCF7*, indicative of a naïve or central memory T cell profile (Supp Fig. 4R, S). More interestingly, factor 8 highlighted a population of cytokine producing cells, with top genes including *IL2, IL21, CCL20* and *IFNG* as well as a large number of costimulatory molecules including *TNFRSF18, 4* and *9* (GITR, OX40 and 4-1BB respectively) (Fig. 3G). A direct calculation of a general cytokine expression score highlighted the same populations (Fig. 3H, Supp Fig. 4X, Methods). This factor largely mapped to CD4 + T cells restimulated with nucleoprotein peptide pools and was observed across multiple patients. Of note, cytokine genes are generally hard to capture in the context of scRNAseq of PBMCs because of their low expression in non-stimulated cells across multiple methods ^17^, but their detection here is likely a reflection of our restimulation approach, since little to no non-stimulated PBMCs scored for this cytokine module.

Across CD8 + cells, factor 1 and 5 captured the bulk of the phenotypic variation observed, with factor 1, also enriched in non-restimulated cytotoxic T cells (Supp Fig. 4A,B), associated with an effector profile (*CD8A, CD8B, GNLY, NKG7, GZMK, GZMA*) while factor 5 also included *CD8A, CD8B* and *GNLY* but did not include any granzymes (Supp Fig. 4I,J). It is also interesting to note that factor 5 encompassed *PTPN22* and *EOMES*, which have both been associated with exhaustion phenotypes that may become more apparent during our restimulation assay (Supp Fig. 4Y). Altogether, our results highlight a broad diversity of T cell phenotypes that could only be uncovered after restimulation but are not directly apparent in PBMCs.

In particular, given the importance of cytokine responses in driving neutralizing antibody responses in a sex-specific manner, we focused on the clusters associated with high cytokine production (TCs 16, 18 and 22) and examined sex-specific differences in gene expression in these cells, as a majority of these cytokine producing cells overlapped with cells enriched in factor 8. As a control, we performed a similar analysis in resting PBMCs from the same subjects, overall capturing a broad diversity of male and female donors in our resting (TCs 0, 1) and activated (TCs 16, 18, 22) clusters (Fig. 3I, Supp Fig. 5A). In the activated group of cells (representing 9 female and 7 male subjects, Fig. 3I, Supp Fig. 5A), we observed a strong enrichment of type I IFN-associated pathways (Supp Fig. 5B) and genes (Fig. 3J) in cells derived from male donors, with *IFITM3, ISG15, MX1, IFITM1, OAS1* and *ISG20* all upregulated in these cells (FDR < 0.1, log2 fold change > 0.4). We also observed an enrichment of other immune associated genes, including *GZMA, CD52, LY6E, CXCR6* and *ICAM3*, altogether suggesting that cytokine producing CD4 + T cells have a distinct gene signature in male subjects. Interestingly, when we performed a similar male/female comparison across resting cells, the number of differentially expressed genes in this comparison was more limited and did not include clear biological pathways. For example, when looking at type I IFN associated genes, we only observed 2 DE genes, with *IFIT1* was upregulated in naïve cells from female subjects and *IFITM3* upregulated in naïve cells from males, thus demonstrating that the enrichment of a type I IFN signature in males is only evident in activated but not naïve T cells.

### Repertoire analysis of SARS-CoV-2 responsive T cells

**Figure 4:**
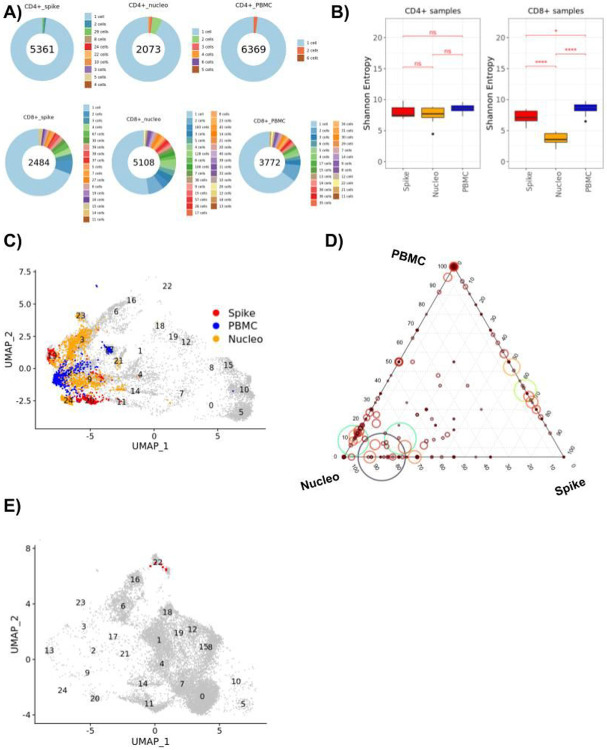
TCR repertoire analysis across steady state and spike or nucleoprotein restimulated T cells **A)** Pie charts displaying the expansion of differently sized CD4+ and CD8+ T cell clones for samples grouped based on their sample type (spike stimulated, nucleoprotein stimulated and PBMC). Numbers in the center of the pie charts stand for the total number of cells of that particular group. A slice in the pie chart with a specific color displays the percentage of cells belonging to clones of size corresponding to this color. Clones that constitute less than 1% of the total population are only available in the pie legend. **B)** Box plots displaying the distribution of the donor specific Shannon entropy values computed based on the CD4+ and CD8+ T cell clones of each donor stratified by the spike, nucleo protein and PBMC samples. **C)** UMAP plot displaying the CD8+ cells, where cells of the expanded clones (n > 20) are highlighted. **D)** Ternary plot of expanded CD8+ clones (clone size > 2) displaying the percentages of PBMC (top corner), Nucleo protein (left corner), and Spike (right corner) cells in the clones. Each clone is depicted as a circle where the circle size and color shows the size of the clone (min =3, max = 221). **E)** Umap plot displaying the CD4+ cells, where the members of a cytokine producing clone in donor256 are highlighted with red.

Another key advantage of analyzing resting and activated T cells simultaneously is to enrich for spike or nucleoprotein TCRs that are likely to be present at a low frequency across all peripheral T cells but enriched by the sorting of spike or nucleoprotein-activated cells. Defining a clonotype based on a common beta chain (including identical VDJ segment usage and CDR3 sequence), we identified several thousand clonotypes across CD4 + and CD8 + T cell populations (Fig. 4A, Methods). CD8 + T cells demonstrated a strikingly stronger clonal expansion than CD4 + T cells, with 25–50% of clones being expanded depending on the condition, versus < 10% for CD4 + T cells. To measure diversity among T cell populations, we then compared the Shannon entropy of PBMCs and restimulated cells and, as expected, observed a significantly lower Shannon entropy for restimulated CD8 + T cells, confirming the significant enrichment of a more restricted pool of clones after restimulation and sorting (Fig. 4B). While there was a trend towards a lower Shannon entropy in restimulated CD4 + T cells as well, this did not reach significance, most likely because of the dominance of single-representative clones in our CD4 + T cell data.

Upon closer inspection of the TCR repertoire, we did not observe a significant difference between the restimulated samples and the PBMC samples for beta chain V and J gene usage (Sup Fig. 6A,B,C,D) and alpha chain J gene usage (Sup Fig. 6E, F). However, we detected a significant increase in TRAV3 and TRAV8 alpha chain V gene usage in both spike and nucleoprotein stimulated cells compared to PBMCs, exclusively in CD8 + T cells (Sup Fig. 6G, H). Given the abundance of expanded CD8 + T cell clones, we decided to focus on the repertoire composition of this population.

The transcriptomic landscape of the CD8 + cells belonging to the expanded clones (size > 20) associated with a more activated and cytotoxic CD8 + profile in all spike, nucleoprotein and PBMC samples, even though the cells formed subclusters based on their sample type (Fig. 4C). We next looked more specifically at the distribution of clones across the PBMCs, spike and nucleoprotein-restimulated samples, with the assumption that the selection of cells activated by a given peptide pool should enrich for clones specific for SARS-CoV2 antigens, and that these clones might also be present at a low frequency in the total PBMCs of convalescent subjects. Indeed, the majority of cells that belonged to expanded clones were associated with the nucleoprotein restimulation condition, with a small degree of overlap with other samples (Fig. 4D). This suggests that our approach has the potential to reveal antigen specific clones by enriching them compared to total PBMC T cells, including clones that are directly associated with cytokine-producing clusters (Fig. 4E).

## Discussion

The analysis of immune responses of individuals naturally exposed to SARS-CoV2 has provided key insights into the development of protective immunity against this virus, and about responses to novel pathogens more generally. Here, we focused on a cohort of individuals that were infected in a similar timeframe through a single spreading event and developed mild disease. In this well-defined group, we focused on the establishment of neutralizing antibody responses and T cell memory. Our results demonstrate a large range of neutralization activity that is not correlated with age and gender. We therefore examined T cell responses as a potential correlate of antibody development, and similarly observed a large diversity of both the magnitude and nature of the response, with IFN-γ generally dominating the response to spike peptides but IL-2, IL-5, IL-17F and TNFa all elicited at significant levels in response to nucleoprotein peptides. Surprisingly, we observed a robust positive association between TNF- responses and neutralizing activity only in male subjects, and no significant association in female subjects.

Sex differences in immune responses have been investigated in many contexts, with female subjects displaying generally higher innate and adaptive immune responses but a higher prevalence of autoimmune disease^18^. Infectious diseases display a more mixed sex-based susceptibility depending on pathogen, but in the case of COVID-19 specifically, the higher susceptibility of male subjects has been well documented ^19^. The analysis of immunological parameters in hospitalized subjects has revealed a number of sex-specific differences in inflammatory cytokines and T cell responses ^20^, although this was not seen in other immunoprofiling studies ^4^ and whether these alterations are directly related to sex or a simply a consequence of differing disease courses has been subject to debate ^21^. Of note, multiple non exclusive mechanisms including differences in mucosa-associated invariant T (MAIT) cells ^22^ and kynurenic acid levels ^23^ have been proposed to explain these differences in susceptibility during acute infection. How these differences translate to the development of memory has remained poorly explored. In our relatively homogeneous cohort (recovered subjects, 6-10 weeks post-mild disease), and after regressing out age, it is interesting to note that we did not observe any significant differences in the magnitude of B and T cell responses, but rather in the orchestration of B-T collaboration during the development of neutralizing antibodies. Long term studies of vaccinated subjects have shown similar antibody titers but lower neutralization activity in males, both early on and 6 months after vaccination ^24^. It is interesting to consider that this may be linked to a distinct contribution of Tfh-dependent and independent B cells, with the latter potentially yielding more diverse but less matured and persistent antibodies in a Tfh-deficient mouse model ^25^.

Single-cell RNA sequencing can identify a large array of T cell receptors and has been employed in the case of SARS-CoV2 to study broad changes in the repertoire ^26^, but provides limited insight into T cell specificities. By contrast, approaches that rely on the enrichment of reactive cells can more directly assess specific responses, without requiring epitope-specific knowledge or tetramers of a matching HLA, as has been performed in acute COVID-19 cohorts ^27^. Here, we leveraged this approach to define both the profile and repertoire of spike and nucleoprotein reactive cells. We were particularly interested in a cluster enriched for cytokine and chemokine production, and identified both sex- and activation status- specific differences within these T cells, suggesting that differences in type I IFN signaling may underlie the sex-specific impact of T cells on neutralizing antibodies. This approach also allowed us to define specific TCRs associated with this population of cytokine producing cells. It is important to note that these studies were performed on subjects infected with the original Wuhan strain, but multiple studies suggest that novel variants, including Omicron, are generally well-recognized by T cells elicited by a previous strain ^28-30^, even in the context of antibody escape.

Altogether, the combination of approaches used sheds light on sex-specific differences in the immune responses to natural SARS-CoV2 infection and uncovers potential mechanistic factors involved. While we focused here on COVID-19, we expect that some of these differences in the orchestration of immune responses may apply to other infectious diseases. A deeper understanding of these processes might both inform vaccine development and provide new clues to the impact of sex on immunity.

## Materials And Methods

### Human Samples

Whole blood (collected in BD Vacutainer K2EDTA tubes) and serum (collected in BD Vacutainer serum tubes) samples from consented subjects recovered from COVID were collected in EDTA tubes at the Brigham and Women’s Hospital, Boston, MA, USA. Results from PCR testing when available, disease and demographic information (Table 1) were collected after blood draws through a RedCap-administered survey. All studies were performed under the IRB protocol number 2020P000849 “Biorepository for Samples from those at increased risk for or infected with SARS-CoV-2.” approved at the Brigham and Women’s Hospital, Boston, MA, USA.

### Serology and neutralization assays

Serum samples were aliquoted from serum tubes and aliquoted for storage at −80°C. Serology was performed at the Broad Institute as described previously^31^. Briefly, MaxiSorp 384-well plates were precoated with 50uL/well of 2.5ug/mL SARS-CoV2 RBD then incubated with 50μL of 1:100 diluted serum samples for 30 min at 37°C. After washing, HRP-anti human IgG and IgM (1:25000 dilution, Bethyl Laboratory) was added to each well for 30 min at RT, before washing and incubation with 40 μl/well of Pierce TMB peroxidase substrate (ThermoFisher). The reaction was stopped by adding 40 μl/well of 0.5 M H2SO4 and the OD was read at 450 and 570 nm on a BioTek Synergy HT.

For neutralization assays with pseudotyped viruses^8,32^, HEK293 cells were cotransfected with psPAX2 (AIDS Resource and Reagent Program), pLenti-CMV Puro-Luc (Addgene), and spike protein expressing pcDNA3.1-SARS CoV-2 SΔCT sequences for the Wuhan strain. Supernatant was collected 48 h posttransfection and mixed with 3-fold serial dilutions of heat-inactivated serum samples. After a 1-hour incubation at 37°C, mixes were used to infect HEK293T-hACE2 cells seeded in 96-well plates at a density of 1.75 × 10^4^ cells per well the previous night. After 48h, cells were lysed in Steady-Glo luciferase assay (Promega) and neutralization titers were defined as the sample dilution at which a 50% or 80% reduction in relative light unit was observed relative to the average of the virus control wells.

For neutralization assays with live SARS-CoV2 virus^31^, serially diluted patient sera samples were mixed with diluted SARS-CoV-2 live virus (D614) and incubated at 37°C for 1 h. Mixes were used to infect Vero E6-TMPRSS2 cells seeded the day prior at 10,000 cells per well in CellCarrier-384-well microplates. After 48h of culture, cells were fixed with 4% PFA for 2h, washed and incubated with a mouse anti-SARS-CoV2 NP antibody (Sino Biological) for 1.5h at RT, followed by Alexa488-conjugated goat-anti-mouse antibody (Jackson ImmunoResearch Labs) for 45 min at RT and nuclear staining with Hoechst 33342 (Thermo Fisher Scientific). Fluorescence imaging was performed using the Opera Phenix^™^ High Content Screening System (Perkin Elmer) and half-maximal inhibitory dilutions (ID50) were determined using a four-parameter, nonlinear curve fitting algorithm, with a total range of 20 to 10,240 (for samples where the minimal dilution did not achieve 50% neutralization or where the maximal dilution exceeded 50% neutralization, respectively).

### PBMC isolation and T-cell restimulation

PBMCs were isolated within 3 hours of blood draw from subjects by density gradient separation using Ficoll-Paque PLUS (VWR- GE Life Sciences) and SepMate tubes (StemCell) according to manufacturer’s instructions. Cells were frozen in Cryostor (StemCell) and stored at −80°C. Either fresh or frozen isolated PBMCs were cultured at 37°C, 5% CO_2_ in RPMI supplemented with 10% fetal bovine serum (FBS), 1% glutamax, 0.5% NEAA and 1% penicillin-streptomycin. Isolated PBMCs from subjects were plated in triplicate at 1 million cells per well in a flat-bottom 96 well plate in 200μL of media. After letting the cells rest for 1 hr, each well was stimulated with 1 μL of 2mg/mL peptide mix for a final concentration of 10 μg/mL of each peptide pool of SARS CoV-2 spike (Miltenyi; cat. # 130-126-701) or nucleoprotein (Miltenyi; cat. # 130-126-699) vs a control with water only. Nucleoprotein peptide mix is a tiled mix of 15mer peptides with an eleven amino acid overlap. Spike peptide mix includes selected immunodominant regions of N-terminal domain and a tiled mix of the C-terminal domain (15mers). Cells were stimulated for 18 hrs, at which time the cell supernatant was saved for further cytokine analysis.

### Flow cytometry and cell sorting

After stimulation of PBMCs with the spike peptide mix, nucleoprotein peptide mix or a control (buffer only) for 18 hours, cells were washed and resuspended in a buffer containing 1% FBS in PBS before staining with surface markers. Cells were first stained with a 1/100 dilution of Fc block (Biolegend). To distinguish cells from different subjects and allow for pooled sequencing, cells were then hashed with the Total-Seq C barcode antibodies at a 1/1000 dilution (TotalSeqC-0251 to 0260, Biolegend). PBMCs were washed 3 times in Cell Staining Buffer (Biolegend, #420201) and combined in pools of up to 8 subjects/8 barcodes. Pools were then stained for 20 min with the following fluorescent antibodies: anti-CD45 (clone HI30), anti-CD3e (clone OKT3), anti-CD4 (clone RPA-T4), anti-CD8 (clone RPA-T8), anti-CD45RO (clone UCHL1), anti-OX40 (clone ACT35) and anti-CD154 (clone 24-31). A Zombie UV (Biolegend) live/dead marker was also utilized. All antibodies were purchased from Thermo-Fischer and used at the recommended dilution. Stained T-cells were sorted into eppendorf tubes based on expression of OX-40 (PE) and CD154 (APC) on a two-way cell sorter (Sony SH800). Only cells restimulated with spike or nucleoprotein peptide pools were sorted, both on CD4+ and CD8+ T-cells that were CD45, CD3 and CD45RO positive from each of the 16 subjects.

### Single cell RNA sequencing

Cells were separated into droplet emulsions using the Chromium Next GEM Single-cell 5’ Solution (v1.1) and the 10x Chromium Controller. 10,000 cells were loaded per channel of the Chromium Next GEM single-cell 5’ (v1.1) Chip G. Following lysis of cells, barcoded mRNA reverse transcription, and cDNA amplification, a 0.6X SPRI cleanup was performed, and the supernatant was set aside for Feature Barcoding library construction as instructed by the Chromium NextGEM single-cell V(D)J v1.1 protocol. A final elution of 45 μL was saved for further construction of libraries. Using the saved supernatant of the 0.6X cDNA cleanup, Feature Barcoding libraries were completed according to the 5’ Next GEM (v1.1) Feature Barcoding library construction methods provided by 10x Genomics. Gene expression and V(D)J libraries were created according to the manufacturer's instruction (10x Genomics).

Gene expression, feature barcoding libraries, and TCR enriched V(D)J libraries were sequenced on a Nextseq500 (Illumina) using a high output 150 cycle flowcell, with the read configuration Read 1: 28 cycles, Read 2: 96 cycles, Index read 1: 8 cycles or sequenced on a HiSeq X (Illumina), using a 150 cycle flowcell with the read configuration: Read 1: 28 cycles, Read 2: 96 cycles, Index read 1: 8 cycles. Feature Barcoding libraries were spiked into the gene expression libraries (at 5% of the sample pool) prior to sequencing.

### Cytokine bead arrays

Cytometric bead arrays (Legendplex Human T-helper cell 12-plex; Biolegend) for IFN-γ, TNF-, IL-2, IL-4, IL-5, IL-6, IL-9, IL-10, IL-13, IL-17A, IL-17F, and IL-22 cytokines were used to quantitatively assess cytokine responses in PBMCs from healthy subjects after *in vitro* stimulation in triplicate with 10ug/mL of the peptide pools of spike and nucleoprotein vs control-treated wells. CBAs were performed on culture supernatants using recommended protocol and analyzed by flow cytometry. Standard curves made from 2-fold dilutions of kit standards were used to interpolate pg/mL concentrations of cytokines in GraphPad Prism 8.1.2 from median PE fluorescence intensities. Median PE fluorescence intensities below the standard curve were set to the theoretical pg/mL detection limit determined by the vendor.

### Multivariate linear regression for evaluating the associations between age, sex, infection status, and processing with cytokine level fold changes upon stimulation

For each cytokine *k* in the list of cytokines containing IL2, IL5, IL6, IL9, IL10, IL17A, IL17F, IL22, IFNg, TNF, let *Y_k_spike_*, *Y_k_nucleo_*, and *Y_k_control_* stand for the measurement levels (in log2 scale) of *k* in spike stimulated, nucleoprotein stimulated and unstimulated control conditions. We assessed the associations between subject age, sex (male or female), infection status (yes or no), and sample processing (fresh or frozen) with fold changes in the level of *k* upon spike (*Y_k_spike_* - *Y_k_control_*) and nucleo (*Y_k_nucleo_* - *Y_k_control_*) stimulations with two separate multivariate linear regression models:

$$Y_{k\_spike}\;\text{-}\;Y_{k\_control}=\beta_{0}+\beta_{1}\text{Age}+\beta_{2}\text{Sex}+\beta_{3}\text{Processing}+\beta_{4}\text{Infected}\;\;Y_{k\_nucleo}\;\text{-}\;Y_{k\_control}=\beta_{0}+\beta_{1}\text{Age}+\beta_{2}\text{Sex}+\beta_{3}\text{Processing}+\beta_{4}\text{Infected}$$

Here ***β***_1_, ***β***_2_, ***β***_3_ and ***β***_4_ are the learned parameters that stand for the effect sizes of changes in age, sex (female over male), sample processing (frozen over fresh) and infected (yes over no) over the fold changes in *k* upon stimulation.

### Preprocessing of scRNA-seq and V(D)J readouts

mRNA and VDJ sequence reads were mapped to the reference human genome GRCh38-3.0.0 with the cloud-based Cumulus workflows ^33^, using the CellRanger 3.0.2 software pipeline. Pooled cells were assigned back to the corresponding donors by using the Python implementation of the Souporcell algorithm ^34^.

### scRNA-seq analysis

For the mRNA data integration, count normalization, dimensionality reduction, clustering, cell scoring, and cluster marker genes detection Seurat R package ^35,36^ was employed. Batch effects, defined as the batch of the sequencing run, were regressed out from the normalized count values with the ComBat algorithm ^37^ implemented in SVA R Package version 3.38.0.

### Preprocessing

Cells which either do not have 10x standard high-quality heavy and light chain V(D)J sequences, or have more than 10% of their transcriptome reads coming from mitochondrial genes were filtered out before the downstream transcriptome analysis. Sets of TRAV, TRAJ, TRBV, TRBJ genes were discarded in all of the downstream analyses.

For the UMI count normalization step, gene expression counts for each cell were divided by the total counts for that cell and multiplied by 10^6^, which was then log-transformed using log1p.

### Dimensionality reduction, graph clustering, and UMAP visualization

Dimensionality reduction was done with PCA identifying the first 50 principal components. For clustering of the cells into expression clusters, a k-nearest neighbor (kNN) graph of the cells was constructed (k=20) using the 50 principal components. Then the Leiden algorithm ^38^ was used to find the clusters of the cells based on the generated kNN graph, with a resolution of 0.1. Expression levels of immunoglobulin genes were discarded during the clustering step. Uniform manifold approximation and projection (UMAP) ^39^ algorithm was run on the first 50 principal factors to obtain the 2D projections of the cells.

### Scoring gene set signatures

Cell specific expression scores of the cell cycle genes and the cytokines were calculated as previously defined in ^40^, where for each gene in the gene-set, 100 genes were randomly selected as control genes.

### Non-negative matrix factorization

The integrated single cell count matrix was non-negative matrix factorized using the Python package Consensus Non-negative Matrix Factorization (cNMF) ^41^. The number of high variance genes used to run the factorization was set to 3000, and the NMF loss function was frobenius. The optimal number of latent factors was chosen based on the identifiability of the independent pathways as determined by gene set enrichment analysis. The genes with significantly high loadings per factor had loadings greater than three interquartile ranges above the 75th percentile. The clusterProfiler R package was used to perform gene set enrichment of the selected genes ^42^.

### Pseudotime Analysis

Pseudotime analyses of the CD4+ and CD8+ T cell populations were performed using the R implementation of Monocle3 ^43^. Root cells were selected based on the transcriptome analyses of the Leiden clusters.

### TCR repertoire analysis

While generating the assembled V(D)J sequences, the 10x Genomics V(D)J contig assembly algorithm (https://support.10xgenomics.com/single-cell-vdj/software/pipelines/latest/algorithms/assembly) accounts for many types of noise specific to scRNA-seq data. Nonetheless, only cells with high-quality V(D)J contig sequences in the beta and alpha chains were chosen. The downstream repertoire analysis excluded cells with more than one high-quality alpha or beta chain sequence (i.e., double expressors). Cells having the same beta chain V and J genes as well as CDR3 nucleotide sequences were assumed to be clonally related. A small fraction of clones contained representatives in both CD4+ and CD8+ populations which might have represented contamination events, in those cases the clone was assigned to the dominant population for further analyses.

## Supplementary Material

Supplement 1

## References

[1] Carvalho T., Krammer F. & Iwasaki A. The first 12 months of COVID-19: a timeline of immunological insights. Nat. Rev. Immunol. 21, 245–256 (2021). 33723416 10.1038/s41577-021-00522-1 PMC7958099

[2] Arunachalam P. S. Systems biological assessment of immunity to mild versus severe COVID-19 infection in humans. Science (80-.). 1220, eabc6261 (2020). 10.1126/science.abc6261 PMC7665312 32788292

[3] Lucas C. Longitudinal immunological analyses reveal inflammatory misfiring in severe COVID-19 patients. Nature 2020. 06.23.20138289 (2020). doi:10.1101/2020.06.23.20138289 PMC7477538 32717743

[4] Mathew D. Deep immune profiling of COVID-19 patients reveals distinct immunotypes with therapeutic implications. Science (80-.). 1209, eabc8511 (2020). 10.1126/science.abc8511 PMC7402624 32669297

[5] Zhang J. Y. Single-cell landscape of immunological responses in patients with COVID-19. Nat. Immunol. 21, 1107–1118 (2020). 32788748 10.1038/s41590-020-0762-x

[6] Kreer C. Longitudinal isolation of potent near-germline SARS-CoV-2-neutralizing antibodies from COVID-19 patients. bioRxiv 2020.06.12.146290 (2020). doi:10.1101/2020.06.12.146290 PMC7355337 32673567

[7] Robbiani D. F. Convergent antibody responses to SARS-CoV-2 in convalescent individuals. Nature 584, 437–442 (2020). 32555388 10.1038/s41586-020-2456-9 PMC7442695

[8] Scheid J. F. B cell genomics behind cross-neutralization of SARS-CoV-2 variants and SARS-CoV. Cell 184, 3205–3221.e24 (2021). 34015271 10.1016/j.cell.2021.04.032 PMC8064835

[9] Ferretti A. P. COVID-19 Patients Form Memory CD8 + T Cells that Recognize a Small Set of Shared Immunodominant Epitopes in SARS-CoV-2. (2020).

[10] Le Bert N. SARS-CoV-2-specific T cell immunity in cases of COVID-19 and SARS, and uninfected controls. Nature (2020). doi:10.1038/s41586-020-2550-z 32668444

[11] Peng Y. Broad and strong memory CD4 + and CD8 + T cells induced by SARS-CoV-2 in UK convalescent COVID-19 patients. bioRxiv (2020). doi:10.1101/2020.06.05.134551 PMC7611020 32887977

[12] Stephenson E. Single-cell multi-omics analysis of the immune response in COVID-19. Nat. Med. 27 (2021). 10.1038/s41591-021-01329-2 PMC8121667 33879890

[13] Schultheiß C. Next-Generation Sequencing of T and B Cell Receptor Repertoires from COVID-19 Patients Showed Signatures Associated with Severity of Disease. Immunity 53, 442–455.e4 (2020). 32668194 10.1016/j.immuni.2020.06.024 PMC7324317

[14] Liao M. Single-cell landscape of bronchoalveolar immune cells in patients with COVID-19. Nat. Med. 26, 842–844 (2020). 32398875 10.1038/s41591-020-0901-9

[15] Mateus J. Selective and cross-reactive SARS-CoV-2 T cell epitopes in unexposed humans. Science (80-.). 370, 89–94 (2020). 10.1126/science.abd3871 PMC7574914 32753554

[16] Rydyznski Moderbacher C. Antigen-Specific Adaptive Immunity to SARS-CoV-2 in Acute COVID-19 and Associations with Age and Disease Severity. Cell 183, 996–1012.e19 (2020). 33010815 10.1016/j.cell.2020.09.038 PMC7494270

[17] Ding J. Systematic comparison of single-cell and single-nucleus RNA-sequencing methods. Nat. Biotechnol. 38, 737–746 (2020). 32341560 10.1038/s41587-020-0465-8 PMC7289686

[18] Klein S. L. & Flanagan K. L. Sex differences in immune responses. Nat. Rev. Immunol. 16, 626–638 (2016). 27546235 10.1038/nri.2016.90

[19] Meng Y. Sex-specific clinical characteristics and prognosis of coronavirus disease-19 infection in Wuhan, China: A retrospective study of 168 severe patients. PLoS Pathog. 16, 1–13 (2020). 10.1371/journal.ppat.1008520 PMC7209966 32343745

[20] Takahashi T. Sex differences in immune responses that underlie COVID-19 disease outcomes. Nature 588, 315–320 (2020). 32846427 10.1038/s41586-020-2700-3 PMC7725931

[21] Shattuck-Heidorn H. A finding of sex similarities rather than differences in COVID-19 outcomes. Nature 597, E7–E9 (2021). 34552251 10.1038/s41586-021-03644-7

[22] Yu C. Mucosal-associated invariant T cell responses differ by sex in COVID-19. Med 2, 755–772.e5 (2021). 33870241 10.1016/j.medj.2021.04.008 PMC8043578

[23] Cai Y. Kynurenic acid may underlie sex-specific immune responses to COVID-19. Sci. Signal. 14, 1–12 (2021). 10.1126/scisignal.abf8483 PMC8432948 34230210

[24] Levin E. G. Waning Immune Humoral Response to BNT162b2 Covid-19 Vaccine over 6 Months. N. Engl. J. Med. 385, e84 (2021). 34614326 10.1056/NEJMoa2114583 PMC8522797

[25] Chen J. S. High-affinity, neutralizing antibodies to SARS-CoV-2 can be made without T follicular helper cells. Sci. Immunol. 5652, eabl5652 (2021). 10.1126/sciimmunol.abl5652 PMC8977051 34914544

[26] Wen W. Immune cell profiling of COVID-19 patients in the recovery stage by single-cell sequencing. Cell Discov. 6, (2020). 10.1038/s41421-020-0168-9 PMC7197635 32377375

[27] Meckiff B. J. Imbalance of Regulatory and Cytotoxic SARS-CoV-2-Reactive CD4 + T Cells in COVID-19. Cell 183, 1340–1353.e16 (2020). 33096020 10.1016/j.cell.2020.10.001 PMC7534589

[28] Keeton R. T cell responses to SARS-CoV-2 spike cross-recognize Omicron. Nature 1–5 (2022). doi:10.1038/s41586-022-04460-3 PMC8930768 35102311

[29] Geers D. SARS-CoV-2 variants of concern partially escape humoral but not T-cell responses in COVID-19 convalescent donors and vaccinees. Sci. Immunol. 6, 1–15 (2021). 10.1126/sciimmunol.abj1750 PMC9268159 34035118

[30] Tarke A. Impact of SARS-CoV-2 variants on the total CD4 + and CD8 + T cell reactivity in infected or vaccinated individuals. Cell Reports Med. 2, 100355 (2021). 10.1016/j.xcrm.2021.100355 PMC8249675 34230917

[31] Di Germanio C. SARS-CoV-2 antibody persistence in COVID-19 convalescent plasma donors: Dependency on assay format and applicability to serosurveillance. Transfusion 61, 2677–2687 (2021). 34121205 10.1111/trf.16555 PMC8447038

[32] Vidal S. J. Correlates of Neutralization against SARS-CoV-2 Variants of Concern by Early Pandemic Sera. J. Virol. 95, (2021). 10.1128/JVI.00404-21 PMC8223959 33893169

[33] Li B. Cumulus provides cloud-based data analysis for large-scale single-cell and single-nucleus RNA-seq. Nat. Methods 17, 793–798 (2020). 32719530 10.1038/s41592-020-0905-x PMC7437817

[34] Heaton H. Souporcell: robust clustering of single-cell RNA-seq data by genotype without reference genotypes. Nat. Methods 17, 615–620 (2020). 32366989 10.1038/s41592-020-0820-1 PMC7617080

[35] Butler A., Hoffman P., Smibert P., Papalexi E. & Satija R. Integrating single-cell transcriptomic data across different conditions, technologies, and species. Nat. Biotechnol. 36, 411–420 (2018). 29608179 10.1038/nbt.4096 PMC6700744

[36] Stuart T. Comprehensive Integration of Single-Cell Data. Cell (2019). doi:10.1016/j.cell.2019.05.031 PMC6687398 31178118

[37] Johnson W. E., Li C. & Rabinovic A. Adjusting batch effects in microarray expression data using empirical Bayes methods. Biostatistics 8, 118–127 (2007). 16632515 10.1093/biostatistics/kxj037

[38] Traag V. A., Waltman L. & van Eck N. J. From Louvain to Leiden: guaranteeing well-connected communities. Sci. Rep. 9, 1–12 (2019). 30914743 10.1038/s41598-019-41695-z PMC6435756

[39] McInnes L., Healy J., Saul N. & Großberger L. UMAP: Uniform Manifold Approximation and Projection. J. Open Source Softw. 3, 861 (2018).

[40] Persson E. K. IRF4 Transcription-Factor-Dependent CD103 + CD11b + Dendritic Cells Drive Mucosal T Helper 17 Cell Differentiation. Immunity 38, 958–969 (2013). 23664832 10.1016/j.immuni.2013.03.009

[41] Kotliar D. Identifying gene expression programs of cell-type identity and cellular activity with single-cell RNA-Seq. Elife 8, 1–26 (2019). 10.7554/eLife.43803 PMC6639075 31282856

[42] Yu G., Wang L. G., Han Y. & He Q. Y. ClusterProfiler: An R package for comparing biological themes among gene clusters. Omi. A J. Integr. Biol. 16, 284–287 (2012). 10.1089/omi.2011.0118 PMC3339379 22455463

[43] Trapnell C. The dynamics and regulators of cell fate decisions are revealed by pseudotemporal ordering of single cells. Nat. Biotechnol. 32, 381–386 (2014). 24658644 10.1038/nbt.2859 PMC4122333

