# Supplementary material for: Functional analyses and single cell immunoprofiling uncover sex-specific differences in SARS-CoV2 immune memory development
Source: Res Sq. 2022 Mar 15:rs.3.rs-1416969. Preprint. [Version 1] doi: 10.21203/rs.3.rs-1416969/v1 (PMC8936123; doi:10.21203/rs.3.rs-1416969/v1)

**Supplementary Figures and figure legends**

**
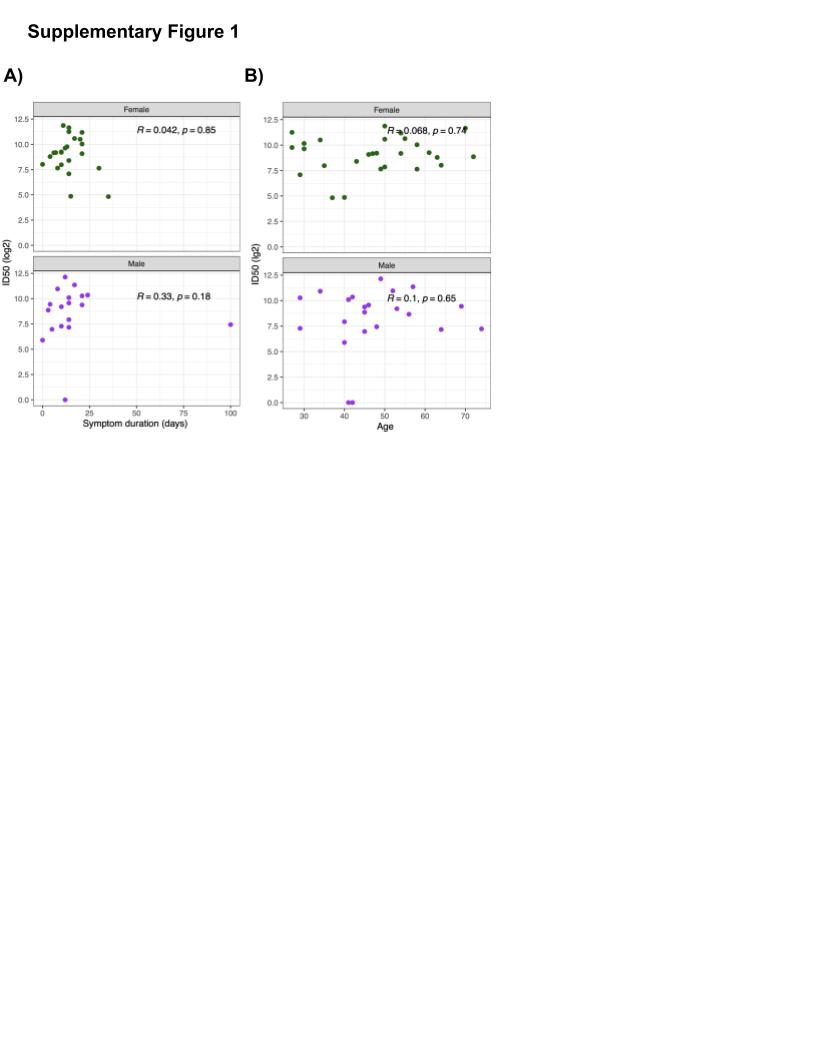
**

**Supplementary Figure 1**

**A)** Scatterplot displaying the relationship between the neutralization titer values (ID50, y-axis) and the symptom duration of the subjects (x-axis) stratified by the gender of the subjects. There is no significant correlation for both genders.**B)** Scatterplot displaying the relationship between the neutralization titer values (ID50, y-axis) and the age of the subjects (x-axis) stratified by the gender of the subjects. There is no significant correlation for both genders.

**
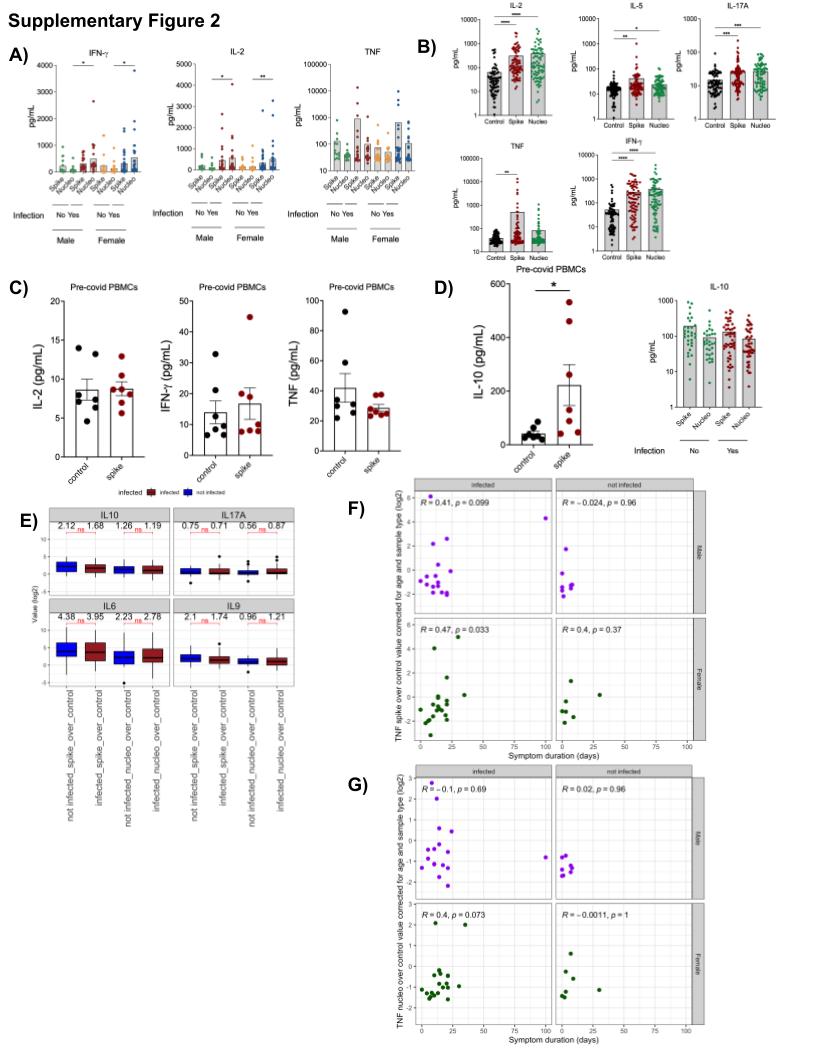
**

**Supplementary Figure 2**

**A)** A plot of concentrations of IFN-γ, IL-2 and TNF measured in the supernatants of PBMCs re-stimulated with either spike or nucleoprotein. On the x-axis, subject PBMC samples are stratified based upon presence of a previous infection with SARS CoV-2 (Yes/No) and the sex of the subject (Male/Female). **B)** Concentrations of the most differential and significant cytokines induced by SARS CoV-2 spike and nucleoprotein peptides; IL-2, IL-5, IL-17A, TNF and IFN-γ. Control samples were treated with peptide vehicle only (water). Representative of 3 replicates per sample. Data in (A) and (B) is representative of 85 individuals. *P ≤ 0.05, **P ≤ 0.01, ***P ≤ 0.001, ****P ≤ 0.0001; one-way ANOVA, Kruskal-Wallis test.

**C)** Plots showing concentrations of known SARS CoV-2 responding cytokines IL-2, IFN-γ, and TNF after re-stimulation with spike and nucleoprotein peptide pools on frozen PBMCs from 7 healthy humans subjects from summer 2019 pre-pandemic. **D)** Plots displaying concentration of IL-10 in pre-pandemic PBMCs (from C) and COVID-recovered PBMCs (from B) after restimulation with the spike and nucleoprotein peptide pools. **E)** Box-plots displaying the distribution of IL10, IL17A, IL6, and IL9 level fold changes upon spike and nucleoprotein stimulation, stratified by the infection status of the patients (red infected, blue non infected). Brackets indicate statistical significance using a one-sided non-parametric Wilcoxon test with *P ≤ 0.05, **P ≤ 0.01, ***P ≤ 0.001, ****P ≤ 0.0001 and n/s indicating no statistical significance. **F)** Scatterplots displaying the correlation between log2 fold change values of TNF after spike **(F)** or nucleoprotein **(G)** restimulation corrected for subject age and sample type (fresh or frozen) (y- axis) and symptom duration (days, x-axis) for male (top row, purple) and female (bottom row, green) subjects stratified by their infection status.

**
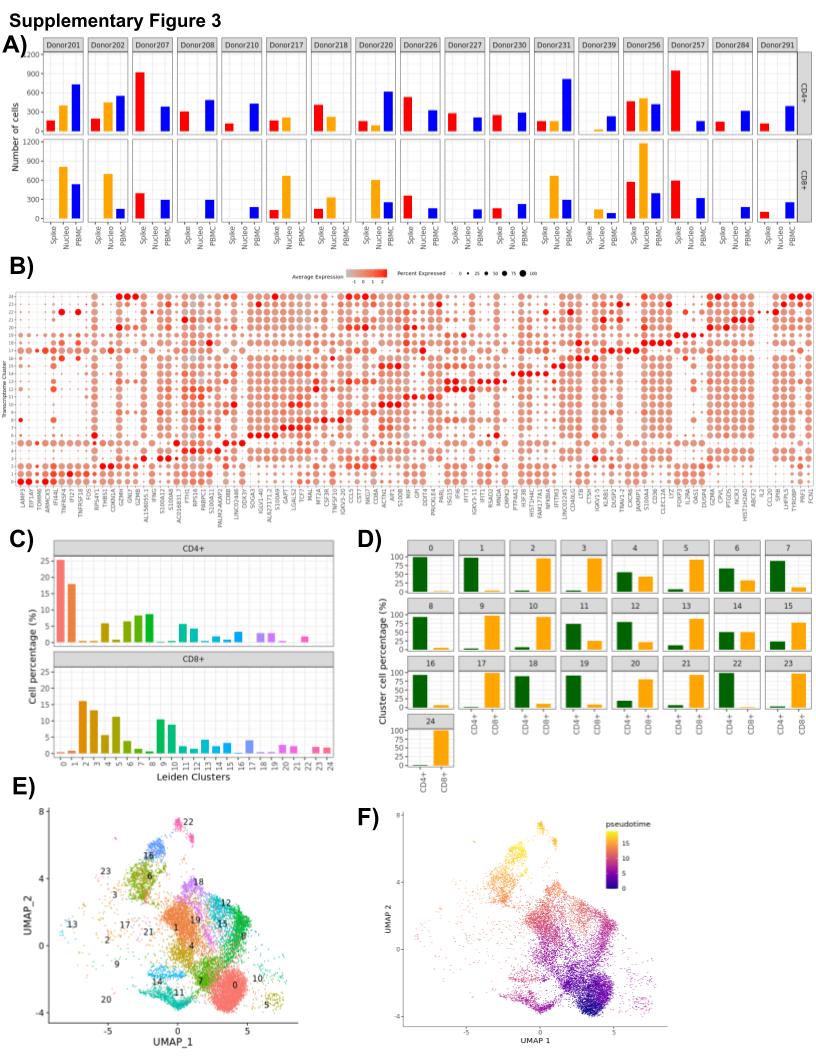
**

**
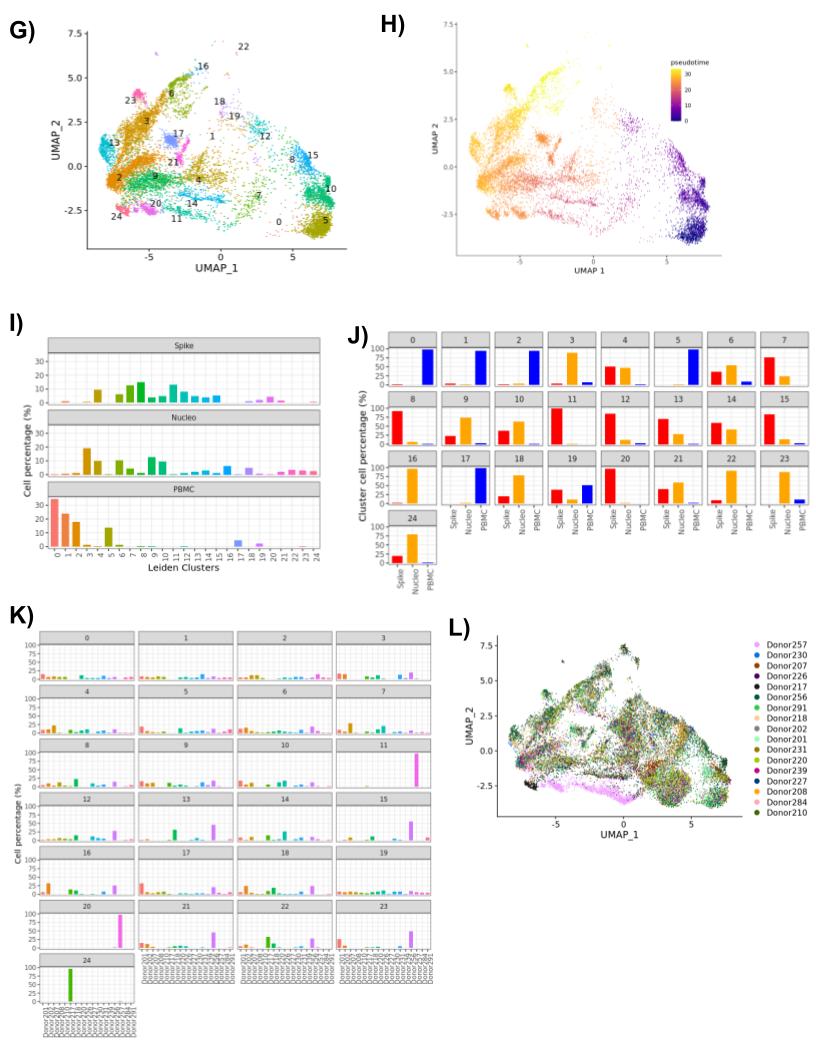
**

**Supplementary Figure 3**

**A)** Bar plot displaying the number of spike restimulated, nucleoprotein restimulated and PBMC CD4+ and CD8+ cells of each of the 17 donors. **B)** Dot plot displaying the expression levels of top 5 marker genes of 24 transcriptome clusters. **C)** Bar plot displaying the distribution of the percentages of the 24 transcriptome clusters in CD4+ and CD8+ T cells. **D)** Bar plot displaying the percentages of CD4+ and CD8+ cells in the 24 transcriptome clusters. **E)** Uniform manifold approximation and projection (UMAP) plot displaying CD4+ annotated T cells, cells are colored based on the clusters they belong to. **F)** UMAP plot displaying CD4+ annotated T cells, cells are colored based on their pseudotime values (Methods). **G)** UMAP plot displaying CD8+ annotated T cells, cells are colored based on the clusters they belong to. **H)** UMAP plot displaying CD8+ annotated T cells, cells are colored based on their pseudotime values (Methods). **I)** Bar plot displaying the distribution of the percentages of the 24 transcriptome clusters in spike restimulated, nucleoprotein restimulated and PBMC cells. **J)** Bar plot displaying the percentages of spike restimulated, nucleoprotein restimulated and PBMC cells in the cell composition of the 24 transcriptome clusters. **K)** Bar plot displaying the percentages of each of the 17 donors in the cell composition of the 24 transcriptome clusters.  **L)**  UMAP plot displaying the clusters on the 2D projections of the cells based on their scRNAseq transcriptome profiles, cells are colored based on the donors they belong to.

**
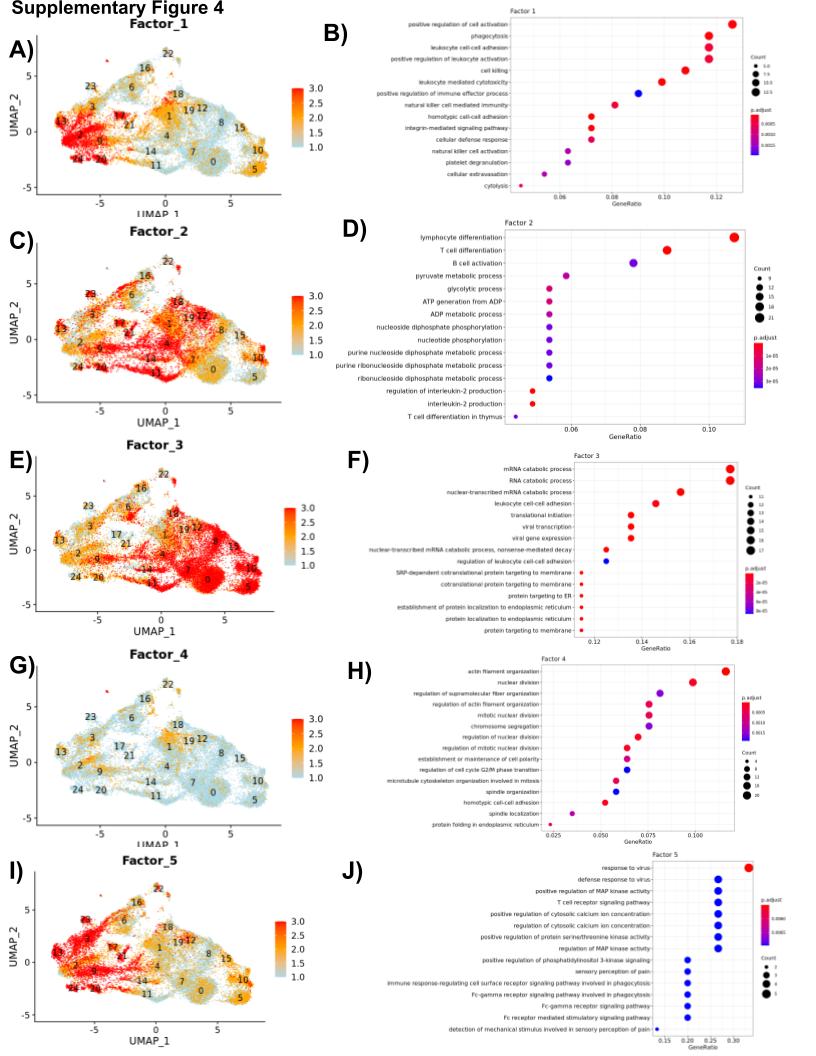
**

**
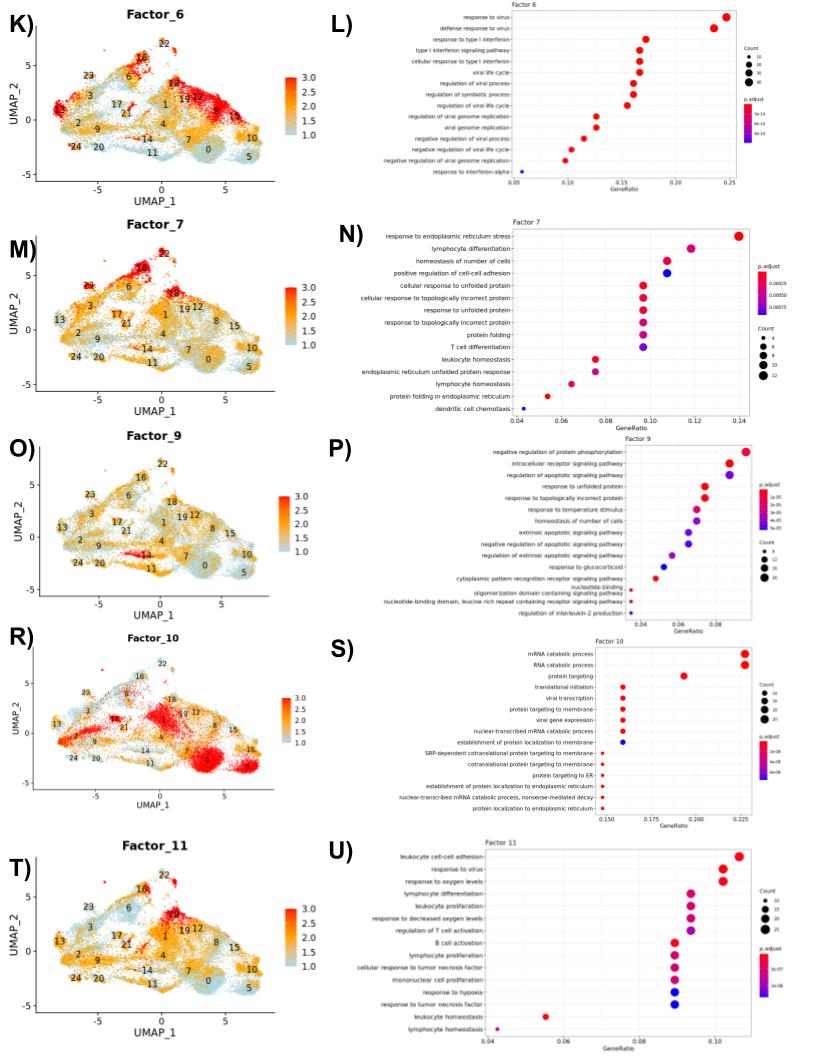
**

**
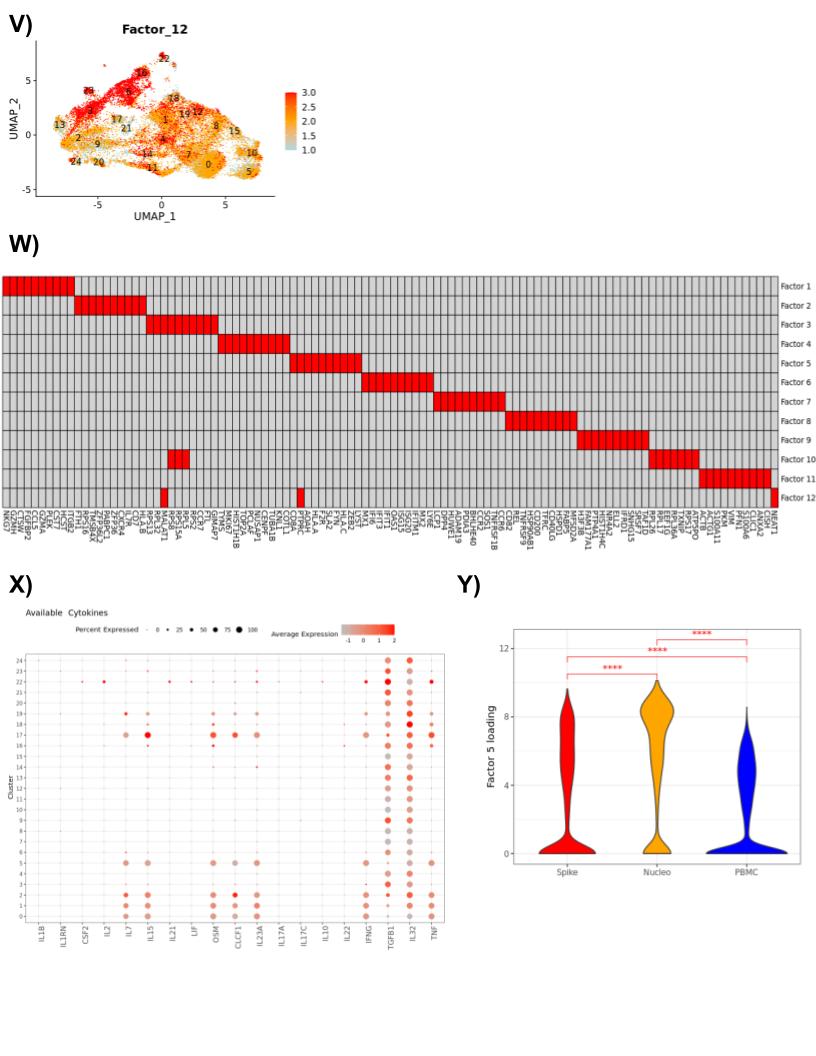
**

**Supplementary Figure 4**

12 latent factors displaying the major axes of variation of the scRNA-seq transcriptome profiles of the spike restimulated, nucleoprotein restimulated and PBMC samples. Plots in **A, C, E, G, I, K, M, O, R, T** and **W** display the UMAP plots where the cells are colored based on the cell factor loadings of Factors 1-12. Plots in **B, D, F, H, J, L, N, P, S**, and **U** display the enriched GO terms of the genes that have high factor loadings (Methods) for each of the Factors 1-11. **W)** Top genes (max 10) with the highest loadings for each of the 12 latent factors that were identified by non-negative matrix factorization of the scRNA-seq gene expression count data. **X)** Dot plot displaying the scaled expression profiles of the cytokines that were captured by the scRNA-seq (x-axis) across the 24 TCs. **Y)** Violin plots displaying the distribution of the Factor 5 loadings (y-axis) of the cells in spike restimulated, nucleoprotein restimulated and PBMC samples (x-axis). Brackets indicate statistical significance using a two-sided non-parametric Wilcoxon test with *P ≤ 0.05, **P ≤ 0.01, ***P ≤ 0.001, ****P ≤ 0.0001 and n/s indicating no statistical significance.

**
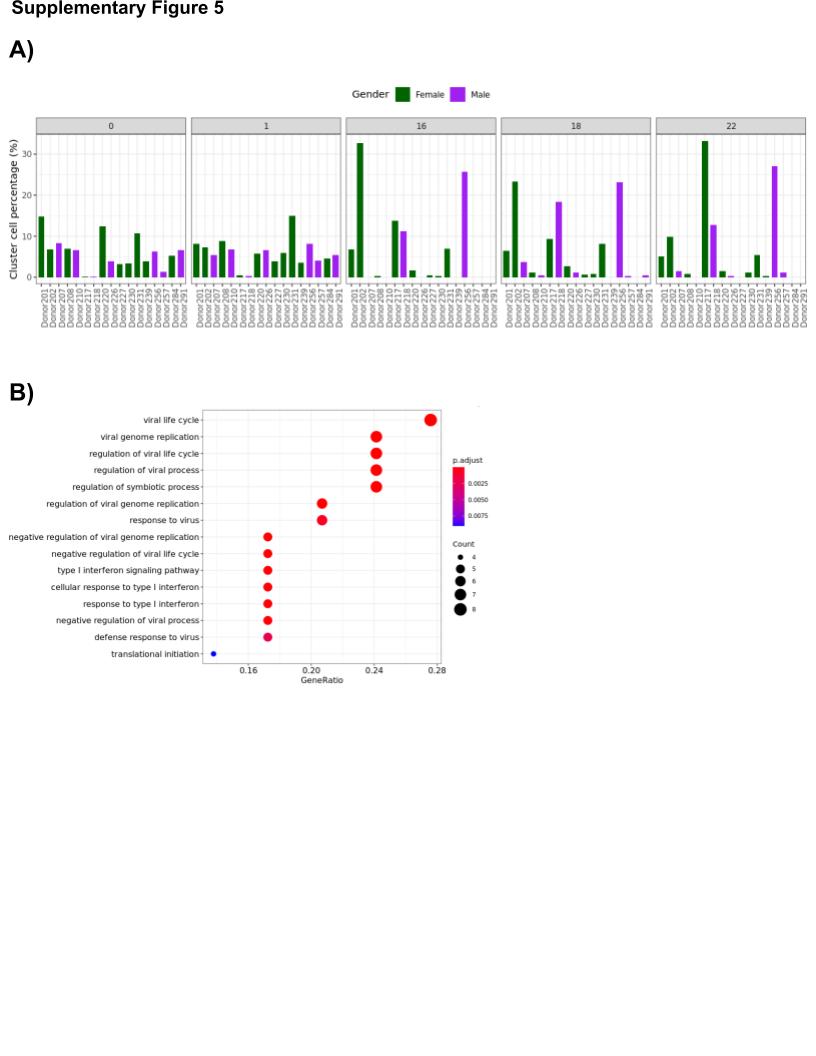
**

**Supplementary Figure 5**

**A)** Bar plot displaying the percentages of each of the male(purple) and female(green) donors in the cell composition of the TCs 0, 1, 16, 18, and 22. **B)** Enriched GO terms for the differentially expressed genes in the group of cells belonging to TCs 16, 18 and 22.

**
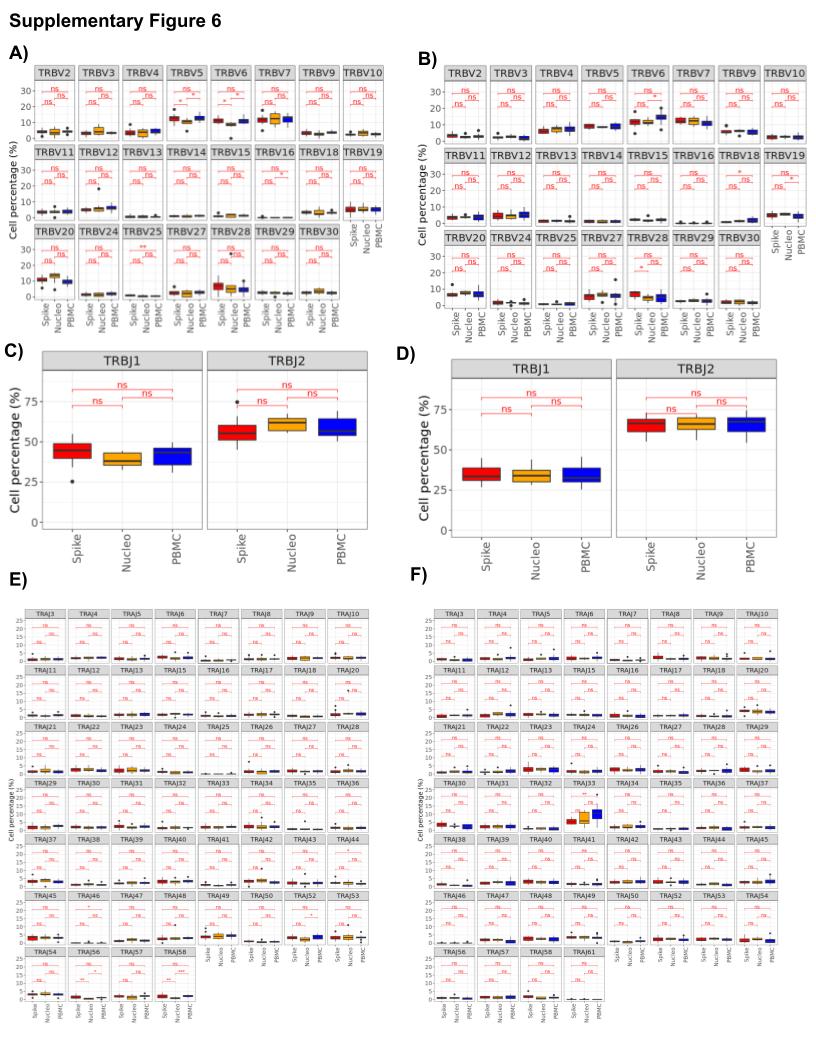
**

**
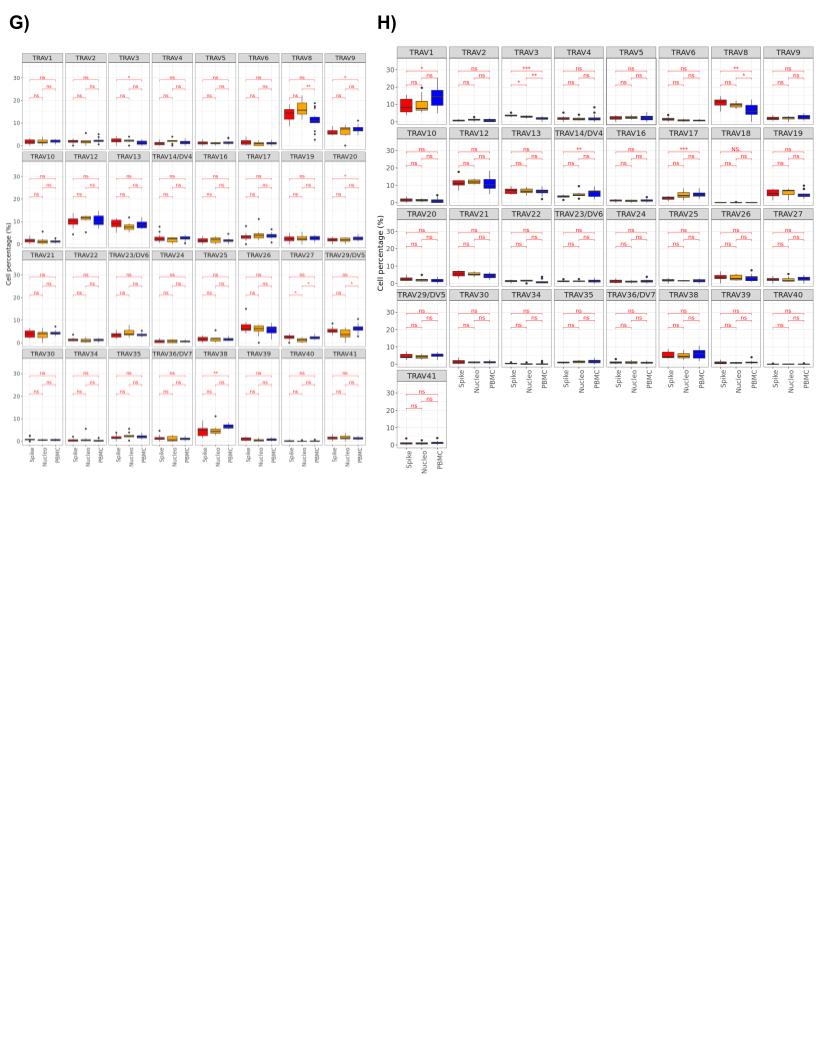
**

**Supplementary Figure 6**

Alpha and beta chain V and J gene usage of CD4+ and CD8+ cells in spike restimulated, nucleoprotein restimulated and PBMC samples. Figures **A**, **C**, **E** and **G** display the boxplots showing the distribution of the donor specific percentage of beta chain - v genes, beta chain - J genes, alpha chain - J genes and alpha chain - V genes in CD4+ cells, stratified by the sample type. Likewise, figures **B**, **D**, **F** and **H** show the distribution of the donor specific percentages of beta chain - v genes, beta chain - J genes, alpha chain - J genes and alpha chain - V genes in CD8+ cells.

Table 1. Clinical and biological characteristics of the cohort


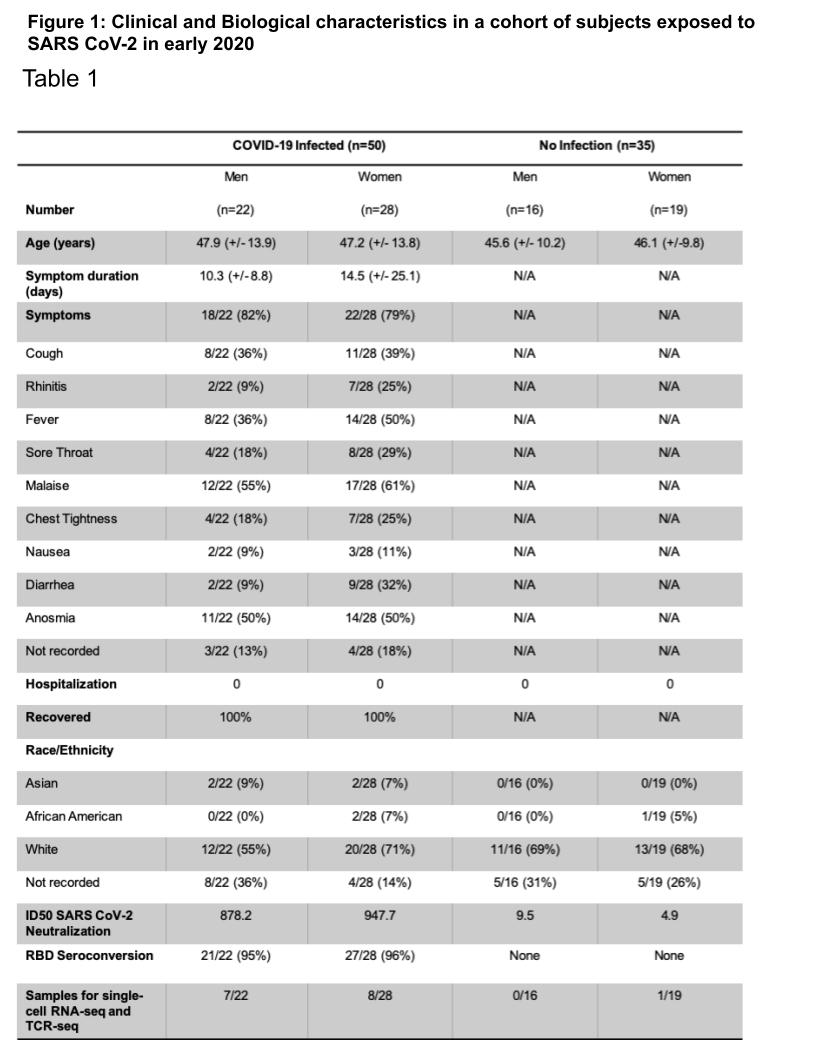

Supplement: Supplement 1 [file 712feca2ddb0a55ece772d84.docx]
